# Experimental Validation of an Analytical Program and a Monte Carlo Simulation for the Computation of the Far Out-of-Field Dose in External Beam Photon Therapy Applied to Pediatric Patients

**DOI:** 10.3389/fonc.2022.882506

**Published:** 2022-07-07

**Authors:** Marijke De Saint-Hubert, Finja Suesselbeck, Fabiano Vasi, Florian Stuckmann, Miguel Rodriguez, Jérémie Dabin, Beate Timmermann, Isabelle Thierry-Chef, Uwe Schneider, Lorenzo Brualla

**Affiliations:** ^1^ Research in Dosimetric Applications, Belgian Nuclear Research Center (SCK CEN), Mol, Belgium; ^2^ Westdeutsches Protonentherapiezentrum Essen (WPE), Essen, Germany; ^3^ Faculty of Mathematics and Science Institute of Physics and Medical Physics, Heinrich-Heine University, Düsseldorf, Germany; ^4^ Physik Institut, Universität Zürich, Zürich, Switzerland; ^5^ Klinikum Fulda GAG, Universitätsmedizin Marburg, Fulda, Germany; ^6^ Hospital Paitilla, Panama City, Panama; ^7^ Instituto de Investigaciones Cient´ıficas y de Alta Tecnología INDICASAT-AIP, Panama City, Panama; ^8^ Medizinische Fakultät, Universität Duisbug-Essen, Essen, Germany; ^9^ West German Cancer Center (WTZ), Essen, Germany; ^10^ Department of Particle Therapy, University Hospital Essen, Essen, Germany; ^11^ Radiation Oncology and Imaging, German Cancer Consortium DKTK, Heidelberg, Germany; ^12^ Radiation Programme, Barcelona Institute of Global Health (ISGlobal), Barcelona, Spain; ^13^ University Pompeu Fabra, Barcelona, Spain; ^14^ CIBER Epidemiología y Salud Pública, Madrid, Spain

**Keywords:** teletherapy, photon, anthropomorphic, pediatric, Monte Carlo, PRIMO, TLD, analytical model

## Abstract

**Background:**

The out-of-the-field absorbed dose affects the probability of primary second radiation-induced cancers. This is particularly relevant in the case of pediatric treatments. There are currently no methods employed in the clinical routine for the computation of dose distributions from stray radiation in radiotherapy. To overcome this limitation in the framework of conventional teletherapy with photon beams, two computational tools have been developed—one based on an analytical approach and another depending on a fast Monte Carlo algorithm. The purpose of this work is to evaluate the accuracy of these approaches by comparison with experimental data obtained from anthropomorphic phantom irradiations.

**Materials and Methods:**

An anthropomorphic phantom representing a 5-year-old child (ATOM, CIRS) was irradiated considering a brain tumor using a Varian TrueBeam linac. Two treatments for the same planned target volume (PTV) were considered, namely, intensity-modulated radiotherapy (IMRT) and volumetric modulated arc therapy (VMAT). In all cases, the irradiation was conducted with a 6-MV energy beam using the flattening filter for a prescribed dose of 3.6 Gy to the PTV. The phantom had natLiF : Mg, Cu, P (MCP-N) thermoluminescent dosimeters (TLDs) in its 180 holes. The uncertainty of the experimental data was around 20%, which was mostly attributed to the MCP-N energy dependence. To calculate the out-of-field dose, an analytical algorithm was implemented to be run from a Varian Eclipse TPS. This algorithm considers that all anatomical structures are filled with water, with the exception of the lungs which are made of air. The fast Monte Carlo code dose planning method was also used for computing the out-of-field dose. It was executed from the dose verification system PRIMO using a phase-space file containing 3x10^9^ histories, reaching an average standard statistical uncertainty of less than 0.2% (coverage factor *k = 1* ) on all voxels scoring more than 50% of the maximum dose. The standard statistical uncertainty of out-of-field voxels in the Monte Carlo simulation did not exceed 5%. For the Monte Carlo simulation the actual chemical composition of the materials used in ATOM, as provided by the manufacturer, was employed.

**Results:**

In the out-of-the-field region, the absorbed dose was on average four orders of magnitude lower than the dose at the PTV. For the two modalities employed, the discrepancy between the central values of the TLDs located in the out-of-the-field region and the corresponding positions in the analytic model were in general less than 40%. The discrepancy in the lung doses was more pronounced for IMRT. The same comparison between the experimental and the Monte Carlo data yielded differences which are, in general, smaller than 20%. It was observed that the VMAT irradiation produces the smallest out-of-the-field dose when compared to IMRT.

**Conclusions:**

The proposed computational methods for the routine calculation of the out-of-the-field dose produce results that are similar, in most cases, with the experimental data. It has been experimentally found that the VMAT irradiation produces the smallest out-of-the-field dose when compared to IMRT for a given PTV.

## 1 Introduction

Therapeutic advances in pediatric oncology have made it possible to increase the survival rates of children with cancer (1). Especially when treating pediatric patients, the protection of surrounding tissue and far-from-the-field tissue is important to prevent the development of radiation-induced second primary cancer (2, 3). Even though second primary malignancies are more likely to appear in high-dose areas, the risk of radiation-induced secondary cancer in lower-dose areas is not negligible (4, 5). Particular attention should be paid in pediatrics since organs are growing with massive cell proliferation (4, 6). Proliferating cells respond sensitively to radiation exposure during cell division (6). Additionally, anatomical structures in pediatric patients are closer in proximity to the treated target, which leads to an increased radiation dose in the same tissue compared to adult patients (4). The cumulative incidence of second primary malignancies is up to 20% of patients treated by radiotherapy (7). The cumulative prevalence rate of long-term sequelae is estimated between 40 and 84% (8, 9). Late effects and late morbidity of cancer treatments become more important, and an improved local tumor control does not have to compromise the protection of patients against long-term effects (4). Studies have shown that pediatric cancer patients have a three- to six-fold increased risk of developing a second primary cancer compared to the general population (5).

It is well known that clinical treatment planning systems (TPS) do not provide an adequate estimation of the out-of-field dose (3, 10–12). Planning computerized tomographies (CT) only include the target volume and organs-at-risk (OARs) in proximity to the treatment field since, for radiation protection purposes and other considerations, they do not cover the full body. Even more important is the fact that algorithms in TPSs are, in general, not conceived for the simulation of the stray radiation far from the irradiated field, and dose measurements in these distant regions are challenging. Consequently, out-of-field dose estimations are limited to regions within the CT volume. Furthermore, the introduction of advanced radiotherapy techniques, such as intensity-modulated radiotherapy (IMRT) and volumetric modulated arc therapy (VMAT), allows a more homogeneous dose delivery to the tumor and potential sparing of the surrounding healthy tissue through spreading of the dose. Nevertheless, for healthy organs further away from the field, only a limited amount of out-of-field dose data evaluating the long-term side effects of these advanced techniques are available. A recent publication has shown that the use of VMAT during craniospinal irradiation (CSI) indicates a reduction of out-of-field doses in most organs (13). Another experimental study from pediatric CSI revealed that the conventional radiotherapy technique, three-dimensional conformal radiotherapy (3D-CRT), resulted in very high doses to a limited number of organs while it was able to spare organs such as the lungs and breast when compared to IMRT and helical tomotherapy (HT). Both IMRT and HT spread the dose over more organs and were able to spare the heart, thyroid, bladder, uterus, and testes when compared to 3D-CRT (14). Finally, another experimental study performed for clinically relevant IMRT and 3D-CRT treatments of the same brain tumor has shown a better reduction of eye and non-target brain doses with 3D-CRT. Moreover, out-of-field doses were comparable for 3D-CRT and IMRT, except for the 3D-CRT irradiation using a mechanical wedge (12). An important limitation of the experimental assessment of out-of-field doses is that the comparison of different techniques that may not be generalized as out-of-field doses will depend on the current practice from the participating centers, applying different objectives and constraints in their dose optimization algorithms. Therefore, the development of methods for the routine calculation of out-of-field doses is a key step in the evaluation and optimization of radiation-induced secondary malignancies in pediatric patients.

Out-of-field dose estimations can be performed by other methods, such as Monte Carlo simulations, analytical methods (15, 16), or direct measurements (11). The purpose of this article is the experimental validation of two algorithms for calculating out-of-the-field absorbed doses. The ultimate goal is to routinely implement these computation techniques in the HARMONIC Consortium, a European project in which 24 clinical and research institutions collaborate in the investigation of radiation-induced primary second malignancies in pediatric patients.

## 2 Materials and Methods

### 2.1 Experimental Setup

Aiming to simulate a realistic photon treatment plan of a brain tumor, a clinically applied treatment plan was transferred to the conditions of the experiment. The corresponding patient should feature a cranial size and shape, which has a reasonable resemblance with the corresponding features of the anthropomorphic phantom. To this end, a 7-year-old female patient with a diffuse midline glioma (WHO grade IV) was selected. The concerned patient was enrolled in the prospective registry study “KiProReg” (German Clinical Trials Register: DRKS-ID: DRKS00005363) after consent was obtained from her legal guardians. This study was approved by the local ethics committee. The patient received a combined radiotherapy and chemotherapy after R3 resection. A dose of 50.4 Gy with 1.8 Gy per fraction was prescribed to the initial PTV, which was located in the cerebellum and had a volume of 195.2 cm^3^.

The experiment was performed using an anthropomorphic phantom ([ATOM, Computerized Imaging Reference Systems (CIRS), Inc, Norfolk, VA, USA] representing a 5-year-old child (type 705D). The phantom consists of tissue equivalent (TE) materials and is predrilled at 180 positions for organ dosimetry. The drilled holes are each filled with TE plugs that keep in position a thermoluminescent detector (TLD).

All irradiations for this article were done with a Varian TrueBeam STx linac operating with flattening filter at a nominal energy of 6 MV. The linac is equipped with a Varian Millennium 120 multileaf collimator.

#### 2.1.1 Treatment Planning

**Figure 1:**
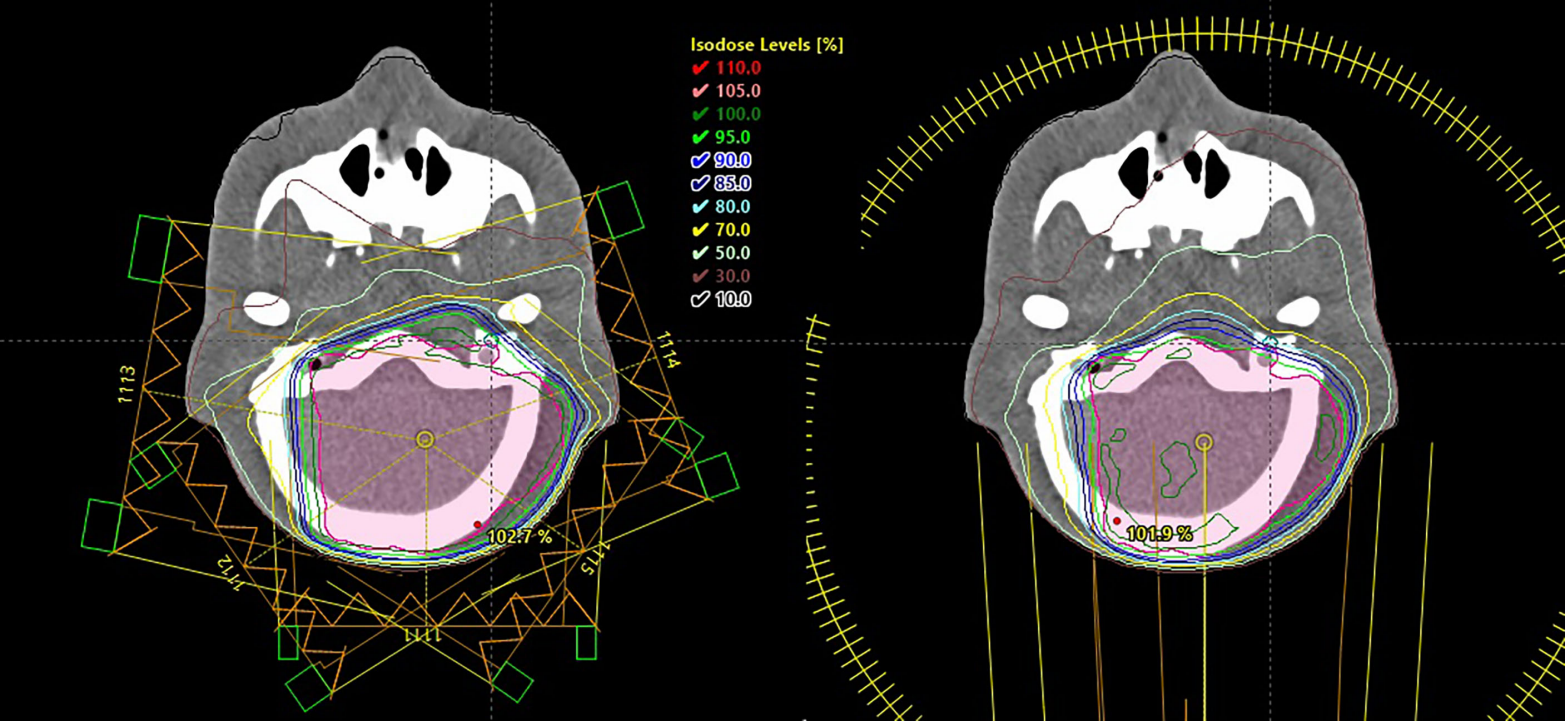
Intensity-modulated radiotherapy (left) and volumetric modulated arc therapy (right) plans showing the isodose lines in the treated volume as computed by the treatment planning system Eclipse.

For treatment planning, the Eclipse External Beam Planning system version 15.6 (Varian Oncology Systems, Palo Alto, CA, USA), using the AAA-algorithm (version 13.6), was employed. Treatment planning was performed using the planning CT of the ATOM phantom. The IMRT plan was calculated with 6-MV photons and consisted of five coplanar and isocentrical fields with beam angles of 70°, 120°, 180°, 235°, and 280°, respectively (see **Figure 1**). In addition, VMAT was planned using two 360° isocentric rotations (see **Figure 1**). The plans were optimized with the photon optimization algorithm PO (Varian Medical Systems, version 13.6). The plans were iteratively optimized over several steps using the constraint V7Gy = 4% for the eyes and V40Gy = 5% and V25Gy = 5% for the left and right cochlea, respectively. A highly weighted general normal tissue objective was used. For comparison purposes of the different treatment plans, it was attempted to reach the predefined goals without further optimization, as it would have been done in a clinical setting. The planning target volume (PTV) was optimized using the prescribed dose as an upper and lower constraint to 0 and 100% of the volume, respectively. The treatment plans were normalized such that at least 95% of the PTV received at least 98% of the prescribed dose. The final plans resulted in 682 and 421 monitor units (MUs) per 1.8 Gy for IMRT and VMAT, respectively.

### 2.2 Dosimetric Measurements

#### 2.2.1 Thermoluminescent Detectors

TLDs were produced by IFJ-PAN (Krakow Poland), namely, natural LiF : Mg, Cu, P (MCP-N) detectors were inserted in 150 out-of-field positions. The delivered dose in the experiments was 3.6 Gy to the PTV as adjusted to the sensitivity of the TLDs. The chosen dose corresponds to 2 fractions of 1.8 Gy of an actual treatment. One set of MCP-N detectors was irradiated with the IMRT plan and another set with the VMAT plan.

Before each exposure, the standard annealing protocol was applied: 10 min at 240°C followed by fast cooling at -10°C inside a temperature-controlled freezer. Following exposure, TLD detectors were read in Thermo Scientific Harshaw 5500 reader following a preheat for 30 min at 120°C to avoid signal fading and low temperature anomalies in the glow curves (17). A heating rate of 10°C/s was used to heat up TLDs up to 255°C. TLDs were calibrated with Co-60 source in terms of kerma “free in air”, *K*_air_. *K*_air_ was then converted to absorbed dose to water (*D*_w_) using the conversion factor *D*_w_/ *K*_air_ = 1.12 as determined by the ratio of mass energy absorption coefficient for water to air for the energy of Co-60 (18). Following normalization to the target dose delivered during treatment of the phantom (3.6 Gy), data were expressed as absorbed dose in water per target dose in units of mGy/Gy.

The distances from the isocenter to the center of each measurement point were calculated using the CT scan of the ATOM phantom. These distances were used for plotting purposes.

#### 2.2.2 TLD Uncertainties

Uncertainties with TLD measurements (coverage factor *k* = 1) were assessed. **Table 1** shows an overview of the uncertainties considered. The included uncertainty sources were dosimeter reproducibility (1.8%), batch reproducibility (1.9%), Co-60 calibration uncertainty (2.4%) as well as background uncertainties which were dependent on the measured dose but remained below 1% (19). From the angular response of MCP-N, previously published by (20), and assuming a uniform angular distribution of radiation, the angular uncertainty of 1% was also included. The uncertainty related to MCP-N photon energy response (17) due to beam softening out-of-field, as described in the study from (21), was also included. Far from the treatment field (at 50 cm off-axis), this study showed a reduced photon energy with an average photon energy of 200 keV. In the present study, a maximum energy dependence of 18% was considered for MCP-N detectors, and following a uniform distribution of the error, we estimated 15% uncertainty on the energy response (*k* = 1).

**Table 1:** Sources of thermoluminescent dosimeter uncertainties (*k* = 1).

Sources of uncertainty	All positions
Dosimeter reproducibility	1.8%
Batch reproducibility	1.9%
Calibration uncertainty	2.4%
Background uncertainty	<1.0%
Angular response	1.0%
Energy response	15.0%
TLD positioning uncertainty	see **Table 2**
Linac uncertainty	2.0%

An investigation on the uncertainty due to the detector positioning within the plug-filled hole was performed for both VMAT and IMRT. Using the Monte Carlo computed (PRIMO) dose distributions, the variation of the dose within 2 mm in the longitudinal axis around each TLD position was assessed. Three regions in the phantom were defined according to their distance to the isocenter, namely, the penumbra (6 to 12 cm), the out-of-field (12 to 40 cm), and the far out-of-field (> 40 cm). The Monte Carlo computed dose for each TLD was classified according to these regions. Afterwards, the computed dose found at each position was compared to the corresponding computed dose 2 mm closer and 2 mm farther from the isocenter along the longitudinal axis of the phantom. The largest relative difference found in this comparison for each region and each treatment modality is reported in **Table 2**. Although the boundaries chosen for the definition of each region are arbitrary, they are related to dose gradient.

**Table 2:** Positioning uncertainties (*k* = 1) for different TLD positions in intensity-modulated radiotherapy (IMRT) and volumetric modulated arc therapy (VMAT) irradiations.

Positioning uncertainty	Penumbra	Out-of-field	Far out-of-field
	6 < d ≤ 12	12 < d ≤ 40	d > 40
IMRT	6.0%	2.0%	<0.1%
VMAT	8.0%	2.5%	<0.1%

Distance *d* to isocenter is expressed in centimeters.

Finally, the uncertainty on the linac dose delivery was estimated to be smaller than 2%. This is the maximum variation allowed by the Swiss authorities. This uncertainty is verified by daily and weekly measurements and eventual adjustments of the linac output if necessary. This 2% value is a very conservative estimate of the uncertainty as, in reality, the linac output is more precise. The treatment plans were delivered for the measurements at the same day to keep the linac output variations minimal.

The combined uncertainty was calculated as the square-root of the linear sum of squared standard uncertainties from **Tables 1**, **3**. The final results of TLD detectors’ uncertainties for both IMRT and VMAT are shown in **Table 4**.

**Table 3:** Total estimated uncertainties (*k* = 1) for different thermoluminescent dosimeter positions in intensity-modulated radiotherapy (IMRT) and volumetric modulated arc therapy (VMAT) irradiations.

Total uncertainty	Penumbra	Out-of-field	Far out-of-field
	6 < d ≤ 12	12 < d ≤ 40	d > 40
IMRT	17%	16%	16%
VMAT	18%	16%	16%

Distance *d* to isocenter is expressed in centimeters.

**Table 4:** Average relative discrepancies between the computed and experimental dose in the three regions defined for both intensity-modulated radiotherapy (IMRT) and volumetric modulated arc therapy (VMAT) irradiations.

Modality, Comparison	Penumbra	Out-of-field	Far out-of-field
	6 < d ≤ 12	12 < d ≤ 40	d > 40
IMRT, PRIMO–experiment	−27%	−13%	−18%
IMRT, analytical–experiment	14%	−14%	−38%
IMRT, analytical–PRIMO	56%	3%	−24%
VMAT, PRIMO–experiment	−27%	−20%	42%
VMAT, analytical–experiment	−44%	−48%	10%
VMAT, analytical–PRIMO	−24%	−35%	−23%

For comparisons with experimental data, the measurements are taken as the reference data set. In the comparisons between the analytical method and PRIMO, the latter is taken as reference.

### 2.3 Analytical Model

A general model to analytically predict the stray dose of radiotherapy plans was applied (15) to the computation of the out-of-field dose in the IMRT and VMAT irradiations of the ATOM phantom. The model concentrates on the three major components of stray dose: patient scatter, collimator scatter, and head leakage. The estimation of the out-of-field dose is based on a mechanistic model for patient scatter, whereas collimator scatter and head leakage were developed using an empirical approach. The parameters of the model were adjusted using measurements of total absorbed dose in simple geometries. The patient scatter contribution requires knowledge about the isocenter dose, the field width, and the field length. The collimator scatter calculation is based on information about the number of MU, the jaw width and length, and the mean multileaf collimator (MLC) length opening. To calculate head leakage, the number of monitor units must be known. The analytical model has been coded to run in the Eclipse (v. 15.6) TPS using the Varian Eclipse Scripting API (16). For this, the CT of the ATOM phantom and the treatment plan is transferred *via* the API into an external software package. Then, the peripheral three-dimensional dose distribution is calculated according to Hauri et al. (15). After that, the TPS dose distribution is fused with the calculated out-of-field dose distribution by determining in the cranial–caudal direction the 5% isodose and replacing the TPS dose with the out-of-field dose for doses smaller than 5% of the TPS dose.

The employed analytical model has a similar conception to that published by (22), with the advantage that the former has been coded as a plugging of the software Eclipse, thus allowing users of this TPS to perform the computation of the peripheral dose during planning. Both models are a substantial evolution of Peridose, published by (23).

The CT of the ATOM phantom consists of 256 × 350 × 256 voxels of size equal to 0.130 × 0.200 × 0.130 cm^3^. The analytical method considers all anatomical structures made of water, with the exception of the lungs which are made of air. For computing the dose to each TLD, each detector was contoured in the CT of the ATOM. Each contoured TLD was considered to be filled of water, independently of its location in the ATOM’s anatomy. In this way, the density and the material composition (water) contained inside each TLD contour are more similar to that of the actual detector which is water equivalent. The algorithm reports all absorbed doses as dose to water.

### 2.4 Monte Carlo Simulation

PRIMO (version 1.0.64.1814) is a Monte Carlo dose verification system that simulates medical linacs and the subsequent absorbed dose computation. The software employs two Monte Carlo engines: the general-purpose radiation transport code PENELOPE 2011 with a modified version of the steering program penEasy and a parallelized version of the fast Monte Carlo code for the simulation of electron–photon showers under radiotherapy conditions called dose planning method (DPM) (24–28).

The dose verification system contains a catalogue of predefined linac geometry files. For the simulations discussed in this article, the Varian C-series (*e*.*g*., Varian Clinac 2100), operating in photon mode with the Varian Millennium 120 MLC, is used. According to the disclosed information from Varian and as it has been experimentally shown (see next paragraph), the geometrical description of the Varian C-series can reproduce the dose distributions produced by the Varian TrueBeam linac operating with flattening filter at a nominal energy of 6 MV, which are the linac and energy employed in all the experiments conducted for this paper. The geometrical description of the Varian C-series contained in PRIMO uses the information provided in the Varian Monte Carlo Package document and the modifications proposed by (29). These documents do not give details about the shielding elements of the linac, which are part of the company’s trade secrets. The geometrical description of the Millennium 120 MLC is done according to the blueprints of the collimator. By following this approach and conducting an accurate transport of radiation through all the collimating and beam-modifying elements of the gantry, it is possible to reproduce the dose distributions conformed by the linac without resorting to non-physical parameters such as the dynamic leaf gap (26, 30, 31). The geometrical description of the MLC is a faithful model of the actual collimator.

PRIMO requires the user to define the characteristics of the pencil electron beam impinging in the bremsstrahlung target through four initial beam parameters, namely, the average energy of the electron beam (< *E* >), the energy full-width at half-maximum (*E*_FWHM_), the beam divergence (*α*), and the FWHM of the circular spot size (*r*_FWHM_). These values are found through a trial-and-error process in which the experimental depth dose and lateral profiles for a 40×40-cm^2^ field irradiating a water phantom are compared with the simulated results for a given set of parameters. The initial beam parameters finally chosen for all IMRT and VMAT simulations were < *E* >= 6.180 MeV, EFWHM = 0.125 MeV, α = 0.000°C, and *r*_FWHM_ = 0.175 cm. With these parameters, simulations of the linac irradiating fields of 40 × 40 cm^2^, 20 × 20 cm^2^, 10 × 10 cm^2^, and 2 × 2 cm^2^ were conducted. The simulated dose profiles in a water phantom were compared to the corresponding experimental profiles *via* the gamma index. The gamma criteria for the evaluations were set to 1%/1 mm. The obtained gamma pass rates were, in all cases, better than 98 and 93% for depth doses and lateral profiles, respectively. When the gamma criteria were relaxed to 2%/2 mm, the gamma pass rates were 100% for all profiles and fields.

PENELOPE and, hence, PRIMO require the user to define a set of radiation transport parameters. The transport parameters *C*_1_ and *C*_2_ were set to 0.02. *C*_1_ determines the mean free path for hard elastic collisions and the cutoff angle to classify elastic events into hard and soft categories. The maximum fractional energy loss allowed within a single step is regulated by the parameter *C*_2_. The cutoff energies *W*_cc_ and *W*_cr_ define the cutoff value for energy losses in inelastic collisions and the cutoff value for bremsstrahlung emission, respectively. For *W*_cc_ and *W*_cr_, the PRIMO default values were kept (both set to 0.2 MeV) (25, 32, 33).

PRIMO allows to tally phase-space files (PSF) at the downstream end of the patient-independent part of the linac, that is, just above the movable jaws. The simulation of the patient-independent part is done with the PENELOPE engine. A sufficiently rich PSF, containing *W*_cc_ and *W*_cr_ histories, was tallied using the chosen initial beam parameters and subsequently employed for all other simulations. The variance-reduction technique of splitting roulette was applied for tallying the PSF (34). The variance-reduction technique of movable skins was applied to all beam-facing surfaces of the linac (35).

DPM was used for the simulation of the radiation transport in the CT of the ATOM phantom. The same CT that was employed for the analytical method was used for the Monte Carlo simulation. The same contoured anatomical structures and contoured TLD positions were also used. For the simulation, the actual chemical composition of the materials used in ATOM, as provided by the manufacturer, was employed. The calibration curve of the CT scanner employed for obtaining the CT image of ATOM was used in PRIMO for converting Hounsfield units to mass density values. It was checked that the yielded density values corresponded to the nominal mass densities reported by the manufacturer of ATOM for each material. Each TLD contour was filled with water in the same way as it was done for the analytical algorithm. PRIMO reports the absorbed dose as dose to medium, which, in the case of the TLDs, was dose to water because the contoured structure enclosing each TLD was filled with water.

The variance-reduction technique of splitting was applied in all simulations starting from the tallied PSF, with a splitting factor of 1,024. This value was chosen by means of a series of preliminary simulations in which the splitting factor was varied, and the simulation efficiency was studied. It was found adequate for not reaching the latent variance of the PSF (36). An Intel(R) Xeon(R) CPU ES-1670 v3 @2.30 GHz (2 processors) with 64-GB RAM was used. All simulations were executed employing 24 logical threads each.

## 3 Results

### 3.1 Comparison of the Computational Methods With the Experiment

The Monte Carlo simulation results reached an average standard statistical uncertainty of less than 0.2% (*k* = 1) on all voxels scoring more than 50% of the maximum dose. The absolute standard statistical uncertainty of out-of-field voxels did not exceed 5%. The simulations of the patient-dependent part of the linac and the CT for IMRT and VMAT took about 7 and 9 days, respectively. These exceedingly long simulation times were required in order to reach the low statistical uncertainty in the voxels located far from the PTV.

**Figure 2:**
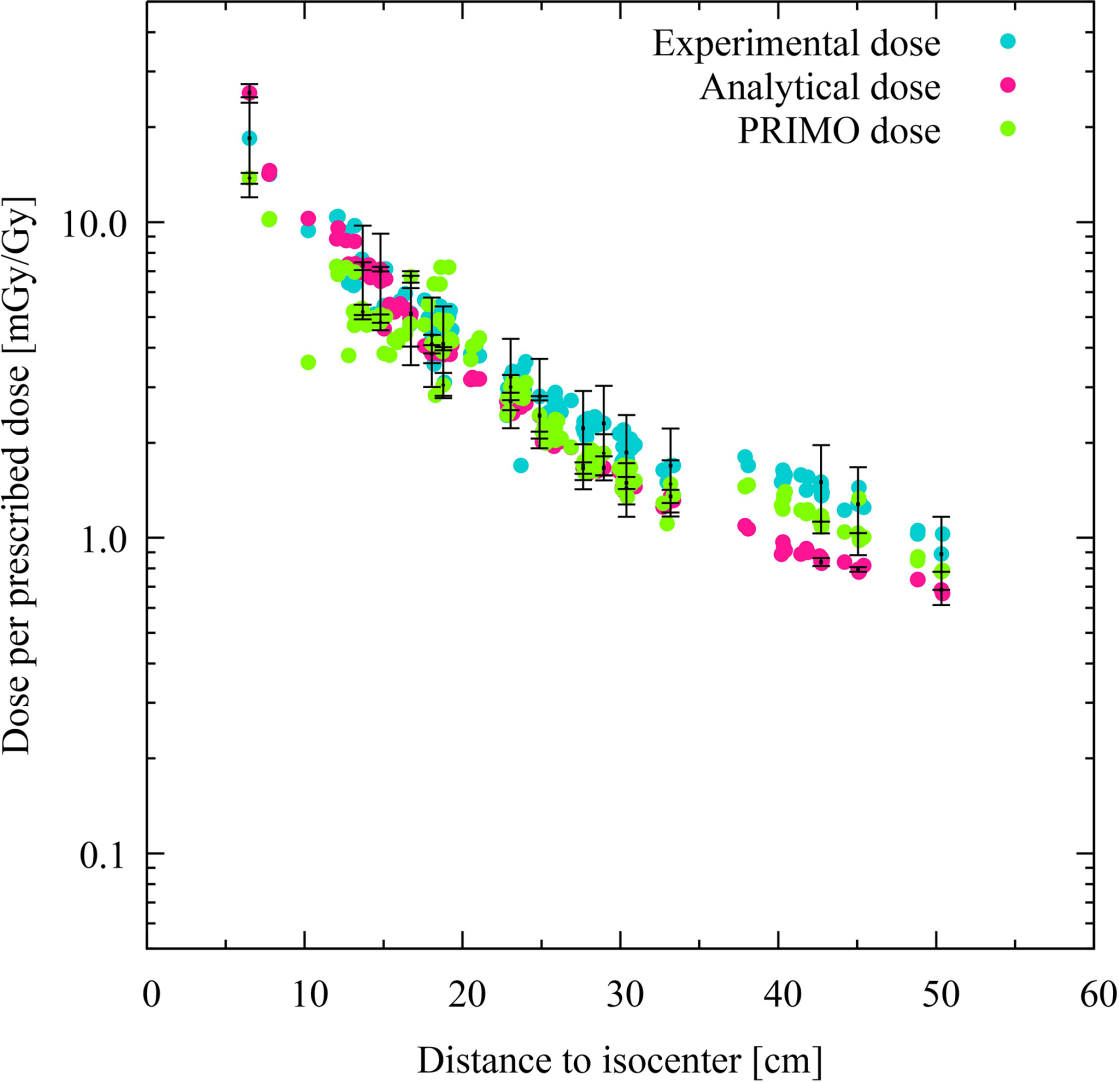
Comparison of experimental, analytical, and simulated data for intensity-modulated radiotherapy. Each colored dot represents one thermoluminescent dosimeter (TLD) position. The distance of the TLD position to the isocenter (in centimeters) is given on the abscissas, while the dose per prescribed dose (mGy/Gy) in logarithmic scale are indicated on the ordinates. The experimental dose distribution is shown with turquoise dots, the analytically calculated absorbed doses with pink dots, and the PRIMO-simulated data with green dots. Statistical uncertainties (*k* = 2) are plotted for every twentieth TLD position.

**Figure 3:**
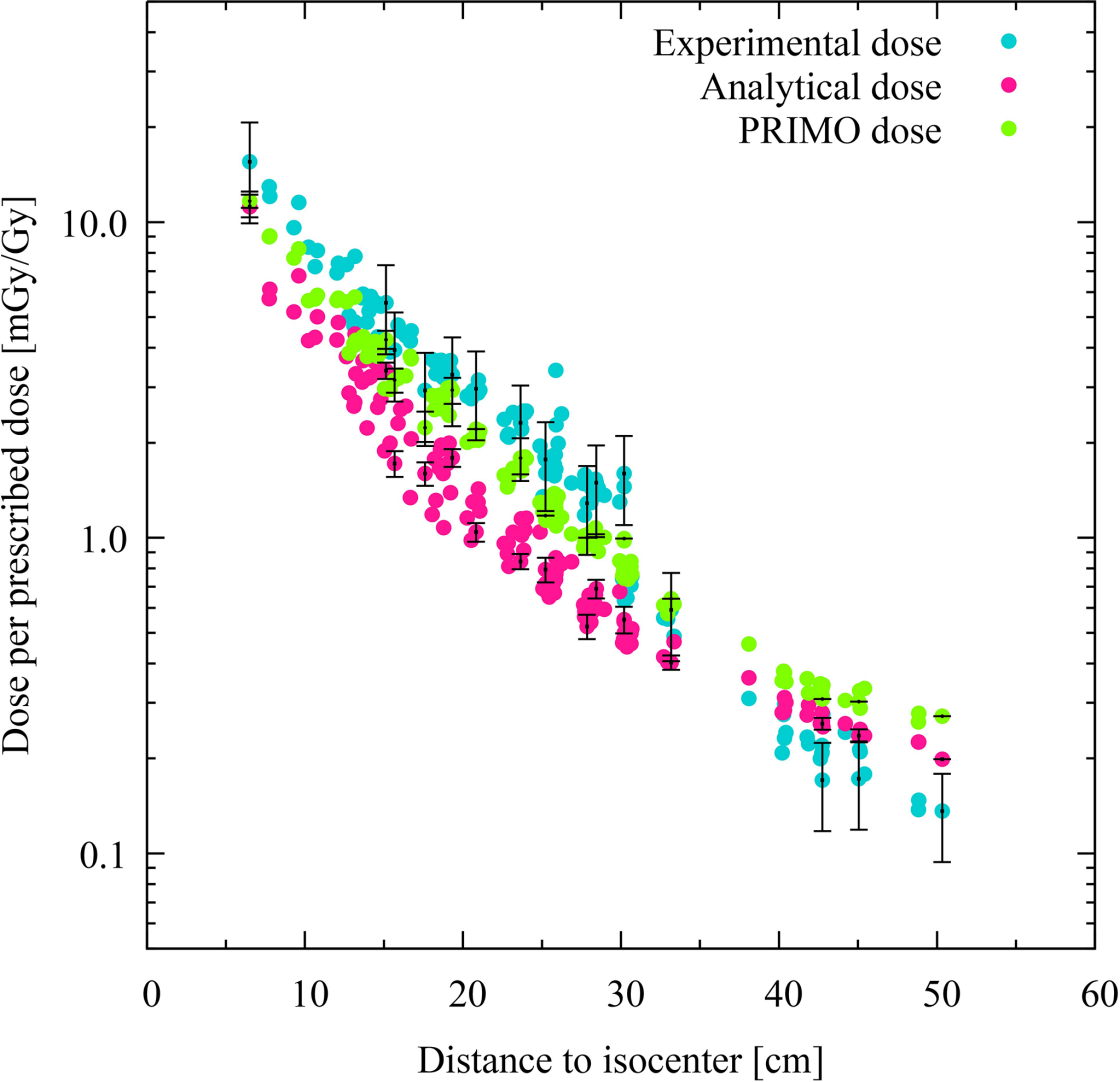
Comparison of experimental, analytical, and simulated data for volumetric modulated arc therapy. Each colored dot represents one thermoluminescent dosimeter (TLD) position. The distance of the TLD position to the isocenter (in centimeters) is given on the abscissas, while the dose per prescribed dose (mGy/Gy) in logarithmic scale are indicated on the ordinates. The experimental dose distribution is shown with turquoise dots, the analytically calculated absorbed doses with pink dots, and the PRIMO-simulated data with green dots. Statistical uncertainties (*k* = 2) are plotted for every twentieth TLD position.

The doses computed at each TLD position of the phantom using the analytical model and the PRIMO simulation are plotted, together with the corresponding experimental data, for the IMRT and VMAT irradiations in **Figures 2**, **3**, respectively. The absorbed doses are presented as a function of the distance to the isocenter.

For the IMRT case, the experimental, analytical, and PRIMO results are compatible within the uncertainty (*k* = 2) in the penumbra and out-of-field regions. In the far out-of-field region, the PRIMO results are compatible with the experimental ones, but the analytical results show a statistically significant deviation from the other two sets of data. The average deviation of the analytical data from the experimental values in the far out-of-field region is of −38% (see **Table 3**).

The discrepancies between the analytical results and the experimental dose are more noticeable in the case of the VMAT irradiation, in which most of the TLD measurements produce results that are not compatible with the analytical data. The average discrepancies between these two data sets are −44% and −48% in the penumbra and the out-of-field region, respectively. The average discrepancy reduces to 10% in the far out-of-field region, although the results are still not compatible. The PRIMO computed results and measurements are compatible within the uncertainty (*k* = 2) for most of the TLD positions in the penumbra and out-of-field region, with average discrepancies of −27% and −20%. However, in the far out-of-field region, the average discrepancies between PRIMO data and experimental data (42%) are larger than those found between the analytical data and the experimental data (10%). In all comparisons with the experimental data, the experiment has been taken as the reference data set. In the comparisons between the analytical results and the PRIMO data, the latter is the reference data set (see **Table 3**).

**Figure 4:**
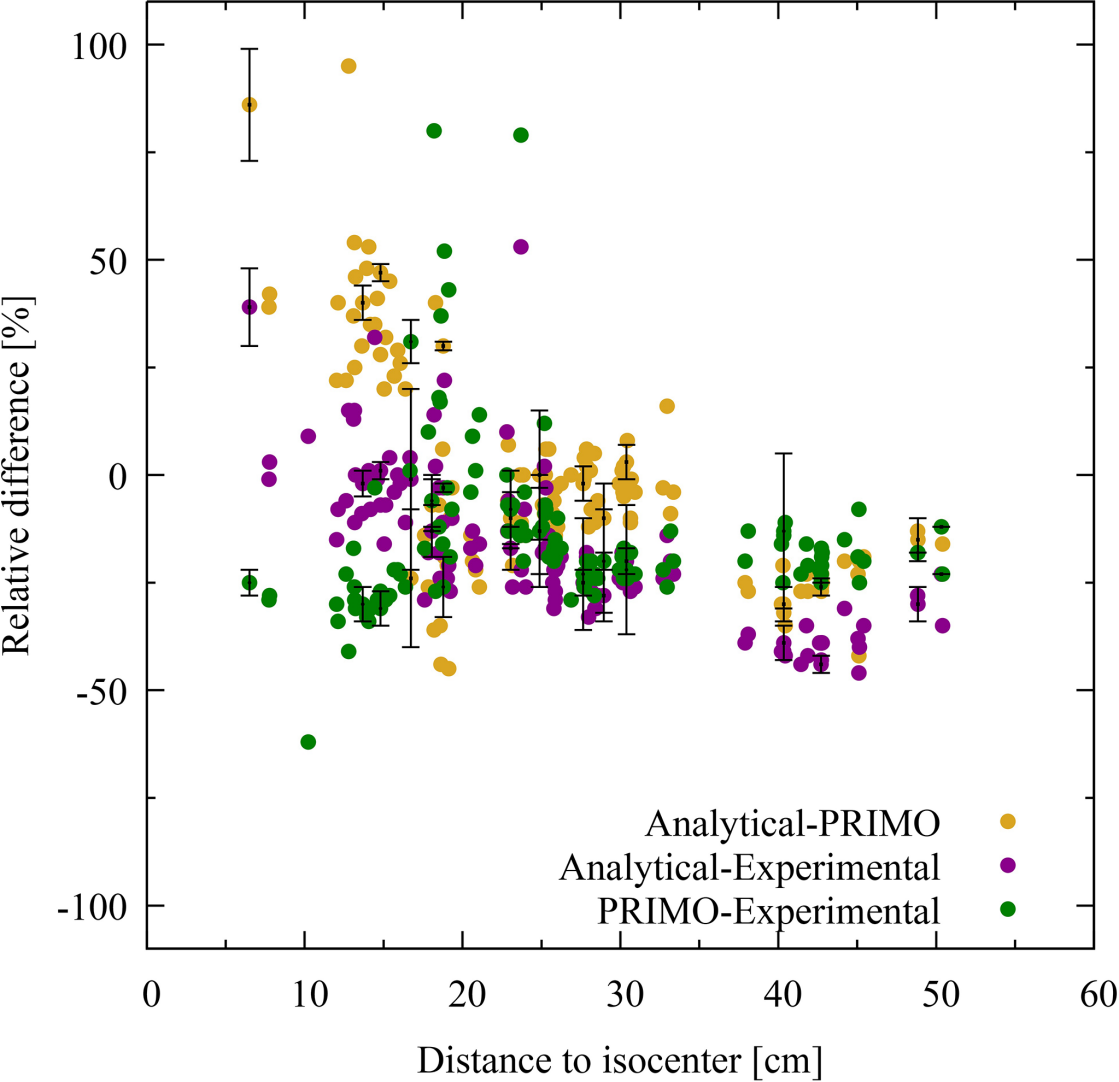
Dose difference for intensity-modulated radiotherapy in percentage for each thermoluminescent dosimeter (TLD) position given with respect to the distance to the isocenter. The difference between PRIMO and analytical data is represented with brown dots, and the PRIMO data set is taken as the reference. The comparison between experimental and analytical data is shown with purple dots and between experimental and PRIMO data with green dots. In these cases, the experimental data is taken as the reference data set. For visual clarity, statistical uncertainty bars (*k* = 2) are shown for every twentieth TLD position.

**Figure 5:**
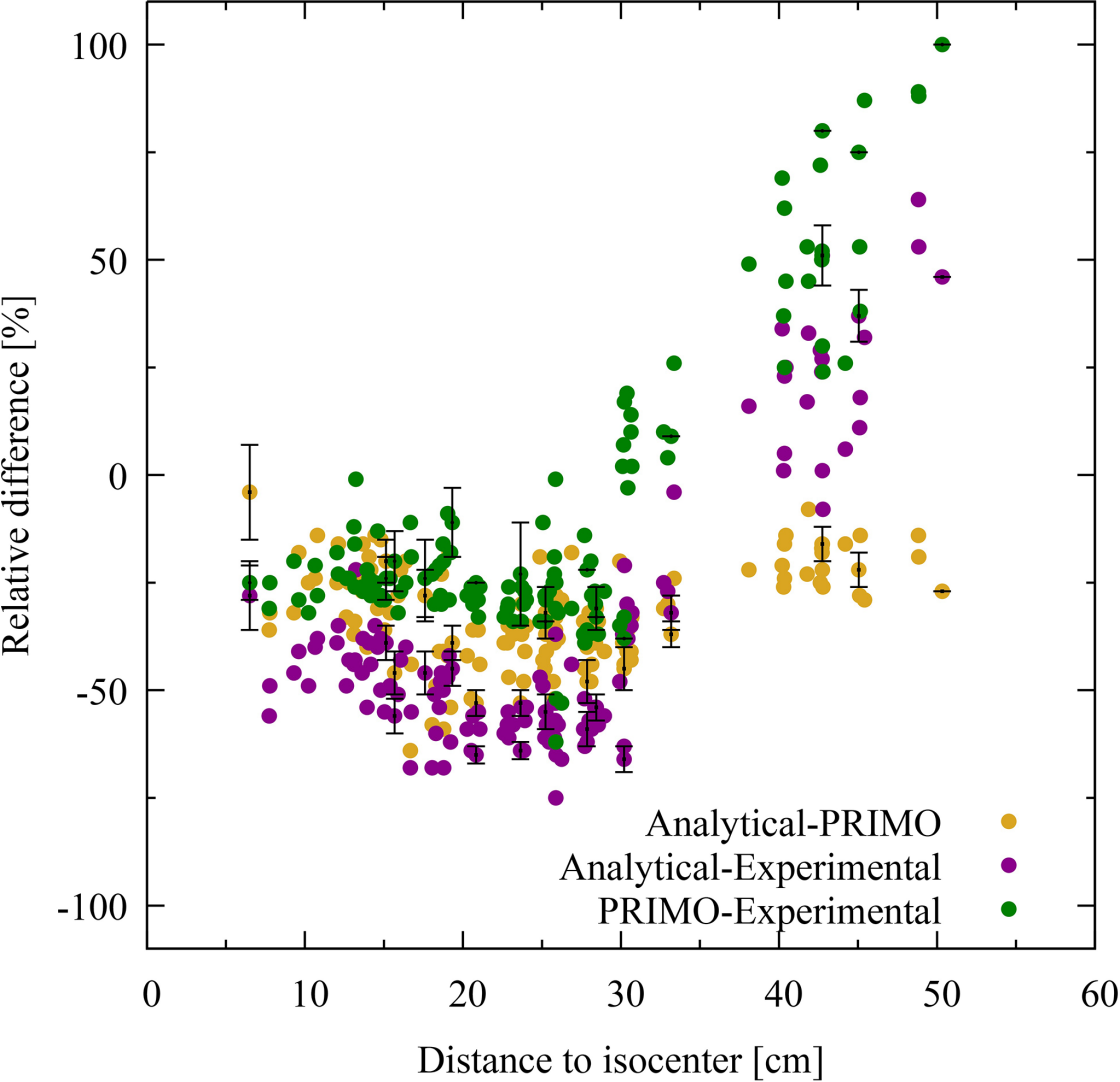
Dose difference for volumetric modulated arc therapy in percentage for each thermoluminescent dosimeter (TLD) position given with respect to the distance to the isocenter. The difference between PRIMO and analytical data is represented with brown dots, and the PRIMO data set is taken as the reference. The comparison between experimental and analytical data is shown with purple dots and between experimental and PRIMO data with green dots. In these cases, the experimental data is taken as the reference data set. For visual clarity, statistical uncertainty bars (*k* = 2) are shown for every twentieth TLD position.

**Figures 4**, **5** show the relative dose difference between the distributions in percentage for each TLD position, with respect to the distance to the isocenter, for the IMRT and VMAT irradiations, respectively. The distance of the TLD position to the isocenter in centimeters is given on the abscissas and the dose difference in percentage is given on the ordinates. **Figure 4**, for the IMRT irradiation, shows that the highest discrepancies appear in the lung region at distances of about 20 cm, with the PRIMO dose being higher than the analytical and the experimental dose. In **Figure 5**, for the VMAT treatment, the largest differences between the three data sets (experimental, analytical, and Monte Carlo) can be observed for TLD locations most far from the treated volume at a distance of 40 to 50 cm.

### 3.2 Comparison Between IMRT and VMAT

**Figure 6:**
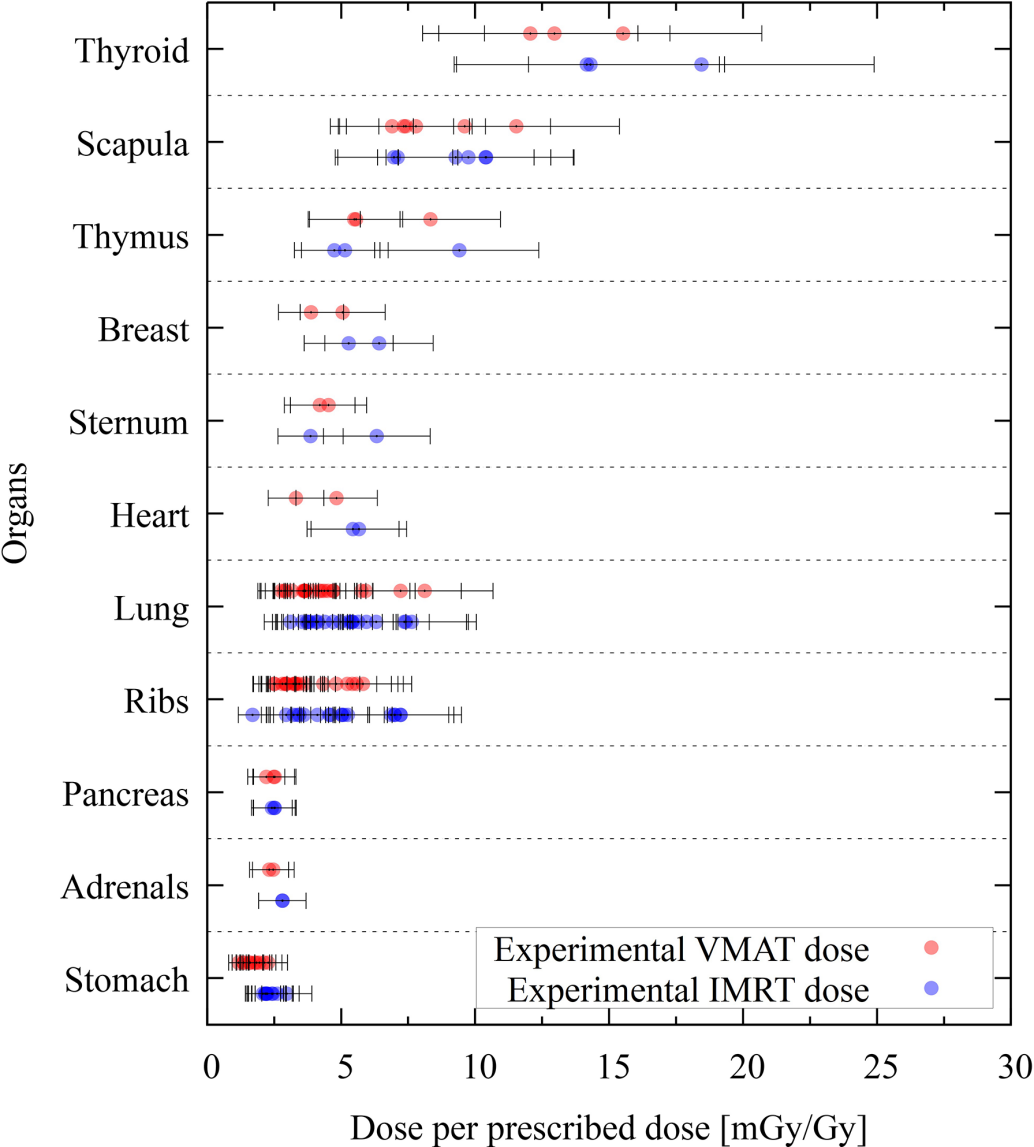
Experimental thermoluminescent dosimeter (TLD) dose values grouped per organ. The dose is given in dose per prescribed dose (mGy/Gy). Absorbed volumetric modulated arc therapy doses are shown with red dots, and intensity-modulated radiotherapy doses are given with blue dots. Statistical uncertainties (*k* = 2) are given for each TLD position.

The IMRT and VMAT modalities can be compared for the given PTV and treatment plan objectives (see Section 2.1.1). **Figure 6** shows the organ dose comparison of IMRT and VMAT for the experimental data. The comparison of the two modalities is presented only through the experimental data since the comparisons obtained through the Monte Carlo or the analytical data yield similar results and the same conclusions. The statistical uncertainties in **Figure 6** are plotted with a coverage factor of *k* = 2. The experimental uncertainties include the positional uncertainty of 2 mm within the hole, which was taken into account by means of the PRIMO computed dose. Each dot in the plot corresponds to a TLD position. The dose per prescribed dose at the PTV is given in mGy/Gy. The thyroid shows the larger dose, with values of around 15 mGy/Gy.

**Figure 7:**
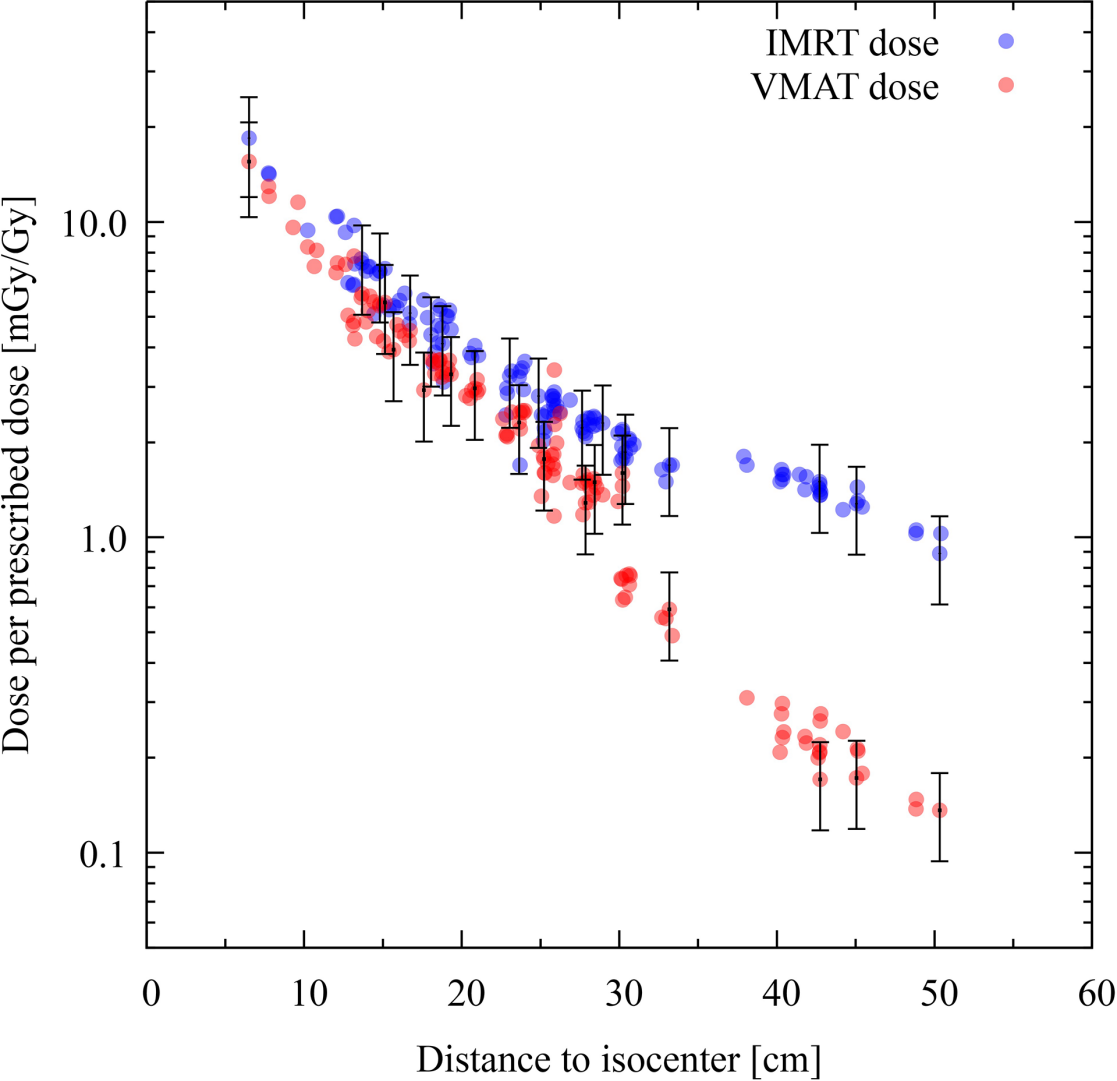
Comparison of intensity-modulated radiotherapy (IMRT) and volumetric modulated arc therapy (VMAT) for experimental data. Each colored dot represents one thermoluminescent dosimeter (TLD) position. The distance of the TLD position to the isocenter (in centimeters) is given on the abscissas, while the ordinates show the dose per prescribed dose (mGy/Gy) in logarithmic scale. The dose distribution for IMRT is shown with blue dots and VMAT with red dots. Statistical uncertainties (*k* = 1) are plotted for every twentieth TLD position.

For all organs, both techniques yield dose values that are similar. However, a clearer picture can be seen if the dose values are plotted as a function to the distance to the isocenter. This is done in **Figure 7**, where the absorbed doses of each TLD position for IMRT and VMAT are plotted in logarithmic scale. It becomes evident that, for positions in the far out-of-field region, IMRT yields an absorbed dose which is about one order of magnitude higher than that from VMAT. The experimental data from both modalities are only compatible in parts of the penumbra and the out-of-field region. The IMRT modality produces an absorbed dose systematically higher than VMAT.

## 4 Discussion

Overall, the two computed distributions feature an acceptable level of agreement with the experimental data for out-of-field considerations and epidemiological concerns considering the limitations of the dose comparison.

### 4.1 Evaluation of PRIMO for Out-of-Field Doses

In general, PRIMO simulations revealed the best agreement to experimental data. It is important to bear in mind that the Monte Carlo Package produced by Varian has the scope of providing researchers with the minimum necessary information for conducting Monte Carlo simulations aimed at reproducing the in-field dose distribution. Concurrently, PRIMO was designed as a dose verification system for radiotherapy, and therefore no specific methods for trying to circumvent the lack of geometrical information on the shielding of the linac have been devised. Still this is the first time PRIMO is used for modeling out-of-field doses, and the results are very promising given the limitations on the geometrical information related to those parts of the linac that have a significant contribution to the stray dose. In general, the agreement reached with the experimental dataset is acceptable and promising for the intended epidemiological studies on second primary cancer. Nevertheless, some discrepancies are observed—for example, during IMRT, the lung dose was higher in PRIMO. Even though PRIMO modeled the lung tissue according to ATOM lung material, the discrepancy could come from TLDs being filled with water (as TLD dose is reported in dose-to-water) instead of LiF. During VMAT, PRIMO doses were compatible except in far out-of-field positions where both analytical and PRIMO doses were significantly higher. This could be explained by the fact that models are not fully correct for far out-of-field positions. In the case of PRIMO, the geometrical description of the linac only includes the parts provided by the manufacturer in the Monte Carlo documentation. The description of the shielding is not provided by the manufacturer, and therefore it is not simulated. The lack of these parts in the Monte Carlo simulation geometry certainly has an influence on the computed stray dose. Moreover, PRIMO does not model all the rooms, and the presence of additional equipment during the experiment is not included in the modeling.

### 4.2 Analytical Model for Out-of-Field Doses

In general, analytical doses underestimate the dose when compared to the experimental and PRIMO doses. This is observed for both IMRT and VMAT. One explanation could come from the fact that the analytical model does not model ATOM materials, but uses water in all tissues, except air in lungs. Therefore, no bone which could explain the lower doses measured in and around bony structures (1.52 g/cm^3^) is modeled. Furthermore, in the case of lung tissue, air is used, which has a lower density than ATOM lung material (0.21 g/cm^3^). The ATOM materials in soft tissue and brain also have slightly higher densities than water at 1.05 and 1.07 g/cm^3^, respectively. Moreover, the analytical method uses, as input for setting up its parameters, dose measurements obtained for specific gantry angles far from the field. It is therefore logical that the lowest average discrepancy observed in the far out-of-field region, with respect to the experimental data, was accountable to the analytical method in the VMAT modality, in which the influence of specific gantry angles is averaged out.

### 4.3 Uncertainties Related to TLD Experimental Data

Positioning uncertainties in the experiment can originate either from the phantom alignment for the irradiation or from filling the TLDs in the organ hole locations of the phantom. The phantom is only aligned by laser marks and not with X-ray control to avoid additional radiation exposure contributing to the TLD dose. The disclaimer of X-ray control is necessary to provide a reasonable comparison between experimental data and analytical or PRIMO data, but the accuracy of the phantom alignment is limited. The impact of positioning uncertainties is larger for in-field and penumbra TLD positions surrounded by larger dose gradients than for TLD positions located far from the treated volume. Another limitation is the uncertainty related to the energy dependence of TLD detectors. The MCP-N type demonstrates a decrease in the relative air kerma response with the decrease of the photon energy down to a local minimum of approximately 0.8 for a photon energy of around 100 keV (17). It has been described in the study from (21) that the beam is softening out-of-field. This study shows a reduced average photon energy of around 200 keV far from the treatment field (at 50 cm off-axis). The mean photon energy, described for open fields and IMRT fields, was dependent on the out-of-field position and also dependent on the field size and tissue heterogeneity. For this reason, it was not possible to use these literature data to correct for the energy dependence of MCP-N detectors, but a calculated uncertainty on the energy dependence based on these literature data, which was on average 15%, was employed instead. The input from simulations to correct experimental data should be handled with caution as experimental data will not be independent from the simulations.

### 4.4 IMRT *Versus* VMAT and Comparison to Published Data

The current study revealed that VMAT irradiation produces results in the smallest out-of-the-field doses when compared to IMRT. Nevertheless, the comparison of different techniques in this study is based on the current practice from the hospital, which makes it difficult to generalize. Indeed results are not only technique dependent but also center dependent, as data might be different if different objectives and constraints are used in the dose optimization algorithms. Previously published experimental data, performed in the same anthropomorphic phantom, also reported on reduced out-of-field doses for VMAT when compared to 3D-CRT (13) for CSI. During brain treatment, the difference between 3D-CRT and IMRT has been shown to be small when not using a mechanical wedge (12). This latter paper reports on out-of-field doses during brain radiotherapy, where the brain tumor was represented by a sphere of diameter 5 cm (planning target volume diameter = 6 cm) located on the left-anterior side of the head (total volume, 113 cm^3^). In the current study, the PTV, which was located in the cerebellum, had a volume of 195.2 cm^3^, so it was slightly larger when compared to the previous study (12). The thyroid doses reported for the 5-year-old phantom was 8.2 mGy/Gy for 3D-CRT, while IMRT resulted in 3.4 mGy/Gy. In our study, the thyroid dose in both modalities is about 15 mGy/Gy (see **Figure 6**). When looking into breast dose, published data showed 3 and 2.6 mGy/Gy for 3D-CRT and IMRT, respectively, while our study revealed doses of 7 and 5 mGy/Gy for IMRT and VMAT, respectively. This comparison reveals an elevated dose in the current study, which can be expected from the increased size of the tumor as well as the different position in the brain. In the published study, the location of the tumor was more cranial (isocenter in slice 3) when compared to our study (isocenter is slice 6). As a result, the organs were closer to the isocenter in our study when compared to (12)—for example, the distance from the isocenter to the thyroid was 7.1 cm in this study *versus* 13.5 cm in (12). When looking into the dose as a function of distance, we can compare our study to the one previously published describing a descriptive and broadly applicable model for stray absorbed dose calculations (37). The model was validated with experimental data using 3D-CRT and for a field size of 10 × 10 cm^2^ at various locations. The modeled doses ranged between 15 mGy/Gy (12 cm) and 0.75 mGy/Gy at 50 cm. The modeled data was underestimated, thus matching nicely the experimental data. These data are comparable to our data (see **Figure 7**). Furthermore, out-of-field doses for different treatment techniques were modeled and, in general, revealed highest doses for Cyberknife, followed by IMRT techniques, while VMAT and Tomotherapy techniques revealed lower doses (37). This is also in line with our study.

### 4.5 Impact on Risk and Epidemiological Studies

Tubiana et al. and Xu et al. have shown that pediatric second primary thyroid cancers are observed following doses as low as 100 mGy (4, 7). Thyroid cancer is the second most frequent second cancer in children associated with a decreasing risk for increasing age at irradiation date and with an increased tendency for female survivors (4, 5). Greater radiation effects for younger children originate from rapid cell proliferation during the development of the thyroid gland (4). Second primary thyroid cancer is recorded after radiation therapy for several primary cancers, including brain tumors (5). The thyroid is located in proximity to the brain, and the results show the thyroid being the organ with the highest absorbed dose outside the field edge. The presented absorbed dose of the thyroid in both modalities is about 15 mGy/Gy (see **Figure 6**). In clinical situations for the studied malignancy, it is common to prescribe 28 fractions of 1.8 Gy, for a total of 50.4 Gy. In a situation like this, the thyroid would receive a cumulative dose of about 750 mGy, widely exceeding the aforementioned 100-mGy value.

Besides an increased risk for thyroid cancer, subsequent secondary primary malignancies of breast, bone, soft tissue, and central nervous system following radiation treatment for childhood cancer have been reported (4, 5). A report from the childhood cancer survivor study relates radiation doses to the skin of more than 1 Gy with an increased risk for basal cell carcinomas (38). The childhood cancer survivor study is a multi-institutional retrospective study analyzing over 14,000 cases of childhood cancer (4). The study also found incidence for second primary neoplasms in survivors of childhood cancer for all primary diagnoses (39, 40). The highest risk for second neoplasms is within 10 years immediately after the first treatment (41). Olsen *et al.* reported that pediatric cancer survivors have a high risk for second malignant neoplasms in the central nervous system, thyroid, and bone (41). The risk to develop neoplasms in the liver, testes, pharynx, intestine, pancreas, and female breast is also higher than in the general population (41). For the lung, uterus, prostate, kidney, and bladder, the risk estimates are close to the risk for the general population (41). Bone sarcoma following childhood cancer are not as frequent as subsequent thyroid cancer but highly fatal (42).

Considerable excess relative risk for stomach cancer was found based on absorbed stomach doses greater than 1 Gy (4). Considering the total dose of 50.4 Gy in the studied treatment, the dose to the stomach would be, for both modalities, around 150 mGy, well below the cited threshold. This is not the case for breast cancer. In the present study, the dose to the breast was found to be about 250 mGy, a value close to 0.5 Gy as found by (4), in which an increased risk appears. Regarding lung cancer, the dose obtained herein is about 300 mGy, while according to (4), lung cancer might occur as a result of radiation therapy after the lungs received scattered doses of around 0.75 Gy. The absorbed doses to the out-of-field organs found in the present study range from 50 mGy (lowest dose in the stomach) to 1 Gy (largest dose in the thyroid). This range is in agreement with that stated by (43) between 10 mGy and 60 Gy for out-of-field organs.

## 5 Conclusion

The proposed computational methods for the calculation of the out-of-field and far out-of-field dose in IMRT and VMAT irradiations produce out-of-field absorbed dose distributions that are adequate for conducting epidemiological studies on radiation-induced second primary cancers. Although PRIMO has been extensively tested as a dose verification system, this is the first time in which the code has been benchmarked against experimental data for far out-of-field absorbed dose distributions. In the case of the analytic model, this is also the first benchmark in which experimental data obtained from measurements on an anthropomorphic phantom have been used. Although there is still room for improvement in both codes, they have shown that they are capable of computing the far out-of-field dose distribution with the accuracy required for epidemiological studies addressed to develop second primary cancer models. Treatment plan optimization taking into account second primary cancer probabilities is an emerging area that is increasingly gaining importance.

The comparison of the out-of-field dose for a given set of planning objectives reveals that the VMAT irradiation produces an out-of-field absorbed dose distribution of up to one order of magnitude lower than IMRT. This phenomenon is known, and it is explained by the geometrical differences in dose delivery between the two techniques and the higher MUs associated to the IMRT treatments with a consequent increment of photon scattering in the MLC. This is a relevant fact when considering the thyroid, which has been identified as an organ with an elevated risk of radiation-induced second primary cancer in brain irradiation of young patients. It is therefore worthy to remark that, despite other elements that must be considered in making a decision, *e*.*g*., the irradiated volume, the lower out-of-field dose to proximal organs produced by VMAT strongly supports it as the modality of choice in cases when radiation-induced second primary cancer is a chief concern.

## Data Availability Statement

The raw data supporting the conclusions of this article will be made available by the authors without undue reservation.

## Author Contributions

MS-H: experimental design, experimental setup, data analysis, and writing of the manuscript. FSu Monte Carlo simulation design, setup and execution, data analysis, and writing of the manuscript. FV: analytical simulation design, setup and execution, data analysis, and writing of the manuscript. FSt experimental setup, writing and review of the manuscript, and Monte Carlo data analysis. MR: Monte Carlo simulation setup, Monte Carlo and analytical data analysis, and writing and review of the manuscript. JD: experimental design and analysis. BT: clinical analysis, writing and review of the manuscript, and methodology. IT-C: epidemiological review, writing and review of the manuscript, and methodology. US: analytical simulation setup, analysis, writing and review of the manuscript, and methodology. LB: conceptualization, supervision, writing of the manuscript, data analysis, and review of the manuscript. All authors contributed to the article and approved the submitted version.

## Funding

The presented research has been funded by the HARMONIC project. The HARMONIC project (Health effects of cArdiac fluoRoscopy and MOderN radIotherapy in paediatriCs) has received funding from the Euratom research and training programme 2014-2018 under grant agreement number 847707. MR acknowledges funding from the Sistema Nacional de Investigación de Panamá. ITC acknowledges support from the Spanish Ministry of Science and Innovation and State Research Agency through the “Centro de Excelencia Severo Ochoa 2019-2023” Program (CEX2018-000806-S) and support from the Generalitat de Catalunya through the CERCA Program.

## Conflict of Interest

The authors declare that the research was conducted in the absence of any commercial or financial relationships that could be construed as a potential conflict of interest.

## Publisher’s Note

All claims expressed in this article are solely those of the authors and do not necessarily represent those of their affiliated organizations, or those of the publisher, the editors and the reviewers. Any product that may be evaluated in this article, or claim that may be made by its manufacturer, is not guaranteed or endorsed by the publisher.
